# Prolonged respiratory failure due to pulmonary embolism in a young woman: a case report

**DOI:** 10.1186/s13256-019-2169-6

**Published:** 2019-07-24

**Authors:** Daniel Tuchscherer, Alexa Hollinger, Jens Bremerich, Martin Siegemund

**Affiliations:** 1 Department of Intensive Care, University Hospital Basel, Spitalstrasse 21, CH-4031 Basel, Switzerland; 2 Department of Radiology and Nuclear Medicine, University Hospital Basel, Spitalstrasse 21, CH-4031 Basel, Switzerland

**Keywords:** Hypalbuminemia, Pulmonary capillary hydrostatic pressure, Pulmonary edema, Respiratory drive, Starling’s equation, Transpulmonary pressure

## Abstract

**Background:**

The pathophysiology of pulmonary edema is generally considered to result from elevated pulmonary capillary hydrostatic pressure due to increased left atrial pressure in consequence of a failing left ventricle.

**Case presentation:**

We present a case of pulmonary edema secondary to severe hypalbuminemia under excessive respiratory drive in a 29-year-old Caucasian woman in respiratory distress with detected bilateral central pulmonary embolism.

**Conclusion:**

In conjunction with severe hypalbuminemia, even the negative intrathoracic pressure swings of respiratory distress may cause pulmonary edema. Detrimental consequences of non-invasive ventilation due to uncontrolled tidal volume and pressure swings need to be considered when treating patients in hypoxemic respiratory failure with low serum albumin.

## Introduction

The pathophysiology of pulmonary edema (PE) is generally considered a result of elevated pulmonary capillary hydrostatic pressure due to increased left atrial pressure in consequence of a failing left ventricle [1], and pulmonary embolism is normally ruled out in the presence of PE. According to Starling’s equation,^1^ fluid flux across the pulmonary capillary wall is determined by the permeability of the vessel wall and net transmural driving pressure, which is a balance of hydrostatic forces that tend to move fluid out of the capillary and colloid osmotic forces that tend to keep fluid in the capillary. PE develops when the amount of fluid crossing the pulmonary capillary endothelium exceeds the capacity of lymph fluid efflux in pulmonary interstitial space [2].

## Case report

A 29-year-old Caucasian woman in respiratory distress was referred for initiation of parenteral nutrition because of chronic malabsorption and malnutrition. She had a history of bariatric gastric bypass surgery 10 years prior with multiple revisions over the following decade due to persisting steatorrhea. Her nutritional history revealed a voluntarily reduced protein intake of 25 g per day. She smoked one pack of cigarettes per day (13 pack years). Alcohol and recreational drugs were negated. Family history was insignificant.

### Physical examination findings

Her blood pressure (BP) was 132/58 mmHg, pulse rate was 120 beats per minute, respiratory rate 28 breaths per minute, and oxygen saturation 80% on room air. Auscultation revealed normal heart sounds and clear lungs. Lower extremity edema was noted and her right calf was tender on palpation. The rest of her clinical examination was unremarkable.

### Diagnostic studies

**Fig. 1:**
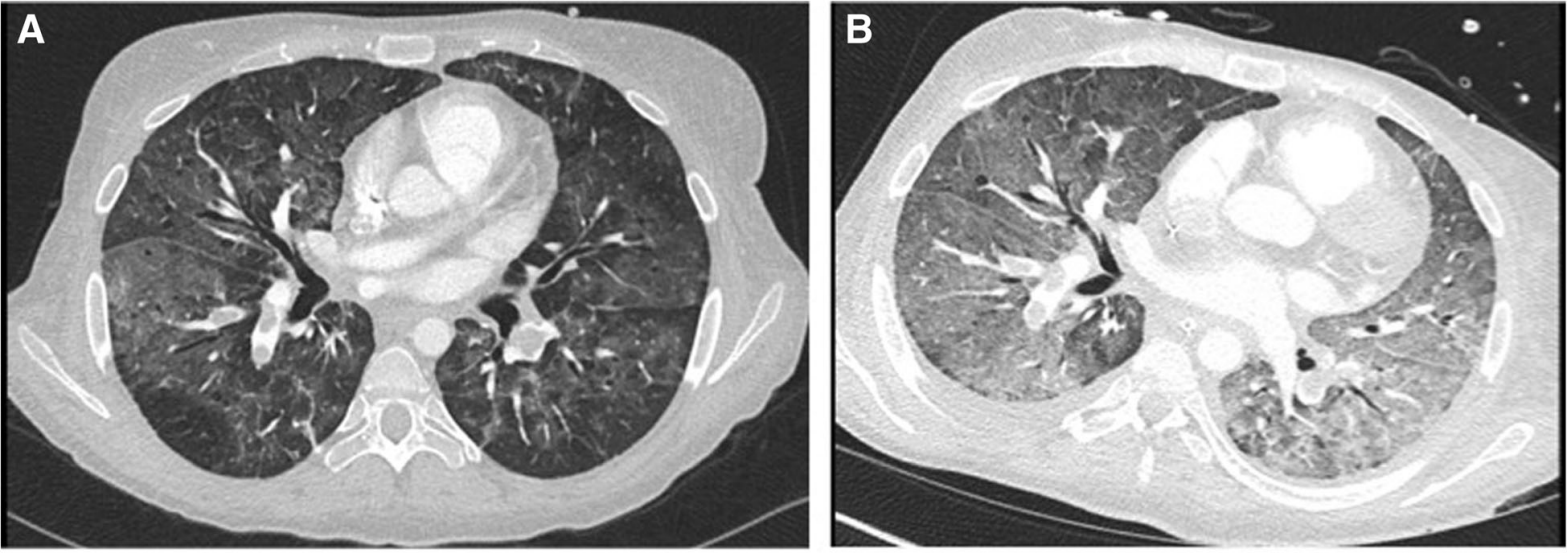
**a** Computed tomography scan of chest on admission showing bilateral central pulmonary embolism and diffuse ground glass opacities. **b** Computed tomography scan of chest after 3 days of non-invasive mechanical ventilation showing profound ground glass opacities

Routine laboratory analyses were normal except for hypalbuminemia (8 g/L, normal range 35–52 g/L), elevated B-natriuretic peptide (BNP; 844 ng/L, normal value < 177 ng/L), and C-reactive protein (CRP; 40 mg/L, normal value < 10 mg/L). Arterial blood gas analysis (BGA) confirmed hypoxemia: partial pressure of oxygen in arterial blood (PaO_2_) of 8 kPa. A computed tomography (CT) scan of her chest revealed bilateral central pulmonary embolism and ground glass opacities (Fig. 1a) [3]. Echocardiography showed dilatation of her right ventricle with normal right ventricular function, while her left ventricle showed systolic D-shaping with normal systolic function [4]. Systolic pulmonary artery pressure (PAP) was estimated at 54 mmHg. Transpulmonary thermodilution and pulse contour cardiac output (Pulsion Medical Systems©) displayed a normal cardiac index (4 L/minute per m^2^), a slightly increased extravascular lung water index (10 ml/kg, normal range 3–7 ml/kg), and a central venous pressure of 17 mmHg. Because of worsening gas exchange over 3 days on non-invasive ventilation (NIV), a repeat CT scan was performed and profound ground glass opacities (Fig. 1b) were noted. Bronchoalveolar lavage (BAL) remained sterile.

### Diagnosis

We diagnosed PE secondary to severe hypalbuminemia under excessive respiratory drive and treated her with thrombolysis, controlled mechanical ventilation, and albumin replacement.

## Discussion

Pulmonary capillary hydrostatic pressure varies with blood flow in the pulmonary circulation and with distribution of pulmonary vascular resistance between precapillary and postcapillary vessels. A pulmonary embolism increases precapillary resistance and hence is usually not associated with increases in pulmonary capillary pressure and development of PE. However, this applies only for areas of the pulmonary vascular bed directly affected by pulmonary embolism [5]. Blood flow is redirected from occluded vessels to open vessels in which increased pressure may be transmitted to the capillary bed. Moreover, pulmonary vascular resistance is directly related to the degree of vascular obstruction and location of the embolus [6, 7].

On the other hand, increased respiratory drive in hypoxemic respiratory failure seems to play a more relevant role in the pathophysiology of PE in the presented case, in addition to hyperadrenergic state causing peripheral vasoconstriction and increase in venous return [8]. Increased venous return may further increase pulmonary blood flow thus contributing to edema. Cyclic changes of alveolar pressures during respiration affect pulmonary capillary hydrostatic pressure. The profound negative intrathoracic pressure swings during respiratory failure lead to a rise of pulmonary transcapillary pressure, the pathophysiologic basis of negative pressure PE [9]. During respiratory efforts, intravascular pressure measured in pulmonary intrathoracic vessels decreases, but to a lesser extent than the esophageal or pleural pressure, resulting in increased transmural pulmonary vascular pressures. Our patient showed high respiratory drive with respiratory rates around 30 breaths per minute and tidal volumes (TVs) of 12.5 ml/kg predicted body weight during the 72 hours of NIV. Increased respiratory drive with the resulting increase of transmural pulmonary capillary pressures probably represents the underlying pathomechanism of patient self-inflicted lung injury due to prolonged application of non-invasive mechanical ventilation in hypoxemic respiratory failure, which seems to be one of the reasons for NIV failure and poor outcome of this patient group. As a consequence of negative pressure swings during experimentally increased respiratory resistance and during increased respiratory drive due to strenuous exercise, lung lymph flow was shown to double instantly. Increased transmural pulmonary vascular pressure in the context of an increased vascular permeability further increases the risk of PE through vascular leakage. Vascular permeability was shown to be increased in vascular injury inflicted by pulmonary embolism.

In addition to the described mechanisms favoring the development of PE, the counteracting determinant (that is, colloid osmotic pressure) was severely reduced in our patient, as serum albumin represents its main determinant. The effect of decreased albumin concentration on the development of PE is well known in experimental physiology. Low colloid osmotic pressure is considered a strong predictor in other lung diseases, such as acute respiratory distress syndrome (ARDS) [10]. The contribution of hypoalbuminemia to PE in clinical practice especially in the context of distinct negative pressure seems comprehensible, constituting hypalbuminemia as a risk factor for cardiogenic PE [11] and increased extravascular lung water in critically ill patients.

Finally, as the major determinant of interstitial fluid clearance is the pressure difference between the pulmonary interstitial space and the venous pressure at the junction of the thoracic duct and jugular vein, lymphatic flow may be significantly reduced in the setting of central pulmonary embolism and the associated rise in central venous pressure.

### Clinical course

Our therapeutic approach targeted all of the components of Starling’s equation. Systemic thrombolysis decreased pulmonary vascular resistance and restored right and left ventricular stroke volume balance. Lung-protective invasive mechanical ventilation minimized patient self-inflicted lung injury by reducing transpulmonary pressure swings and transmural pulmonary vascular pressure. Oncotic pressure was normalized by albumin administration and diuretic therapy. The alveolar PE slowly but continuously abated clinically and radiologically. After 12 days of invasive mechanical ventilation, our patient was discharged to the ward and transferred to a rehabilitation clinic 1 month after admission. On 6-month follow-up, she had fully recovered with normal static and dynamic lung volumes, mildly reduced carbon monoxide (CO) diffusion capacity, normal arterial BGA, as well as normal 6-minute-walking test and echocardiography.

## Conclusion

Negative pressure PE is another well-described etiology of PE [7], possibly of importance in severe acute respiratory failure. In conjunction with severe hypalbuminemia, even the negative intrathoracic pressure swings of respiratory distress may cause PE. Part of the mechanisms underlying patient self-inflicted lung injury may be excessive respiratory drive with resulting increases in pulmonary transmural vascular pressures. Detrimental consequences of NIV due to uncontrolled TV and pressure swings [12] need to be considered when treating patients in hypoxemic respiratory failure with low serum albumin.

## Data Availability

The datasets used and analyzed during the current study available from the corresponding author on reasonable request. The clinical data are stored electronically in the intensive care clinical information system software (MetaVision, iMDsoft®) provided in the intensive care units of the University Hospital Basel.

## Abbreviations

ARDS: Acute respiratory distress syndrome
BAL: Bronchoalveolar lavage
BGA: Blood gas analysis
BNP: B-natriuretic peptide
BP: Blood pressure
CO: Carbon monoxide
CRP: C-reactive protein
CT: Computed tomography scan
NIV: Non-invasive ventilation
PaO_2_: Partial pressure of oxygen in arterial blood
PAP: Pulmonary artery pressure
PE: Pulmonary edema
TV: Tidal volume

## Footnotes

1 Q = Kf [(P_c_–P_i_) - K_π_ (π_c_-π_i_)] Starling’s equation, where Q is the net fluid flow across the pulmonary capillary wall, Kf is the filtration coefficient (a measure of the permeability of the pulmonary capillary wall to fluid flux across it), K_π_ is the oncotic reflection coefficient (a measure of the pulmonary capillary wall resistance to protein movement), P_c_ is the pulmonary capillary hydrostatic pressure, P_i_ is the pulmonary interstitial hydrostatic pressure, π_c_ is the capillary oncotic pressure, and π_i_ is the interstitial oncotic pressure
